# Supplementary material for: The interactome of intact mitochondria by cross-linking mass spectrometry provides evidence for coexisting respiratory supercomplexes
Source: Mol Cell Proteomics. 2017 Dec 8;17(2):216–32. doi: 10.1074/mcp.RA117.000470 (PMC5795388; doi:10.1074/mcp.RA117.000470)
Supplement: Supplemental Data [file supp_RA117.000470_133922_0_supp_23978_fzffwf.zip › spectra_annotation/mito_spectra_annotation/51-1-3-1-1-1.pdf]

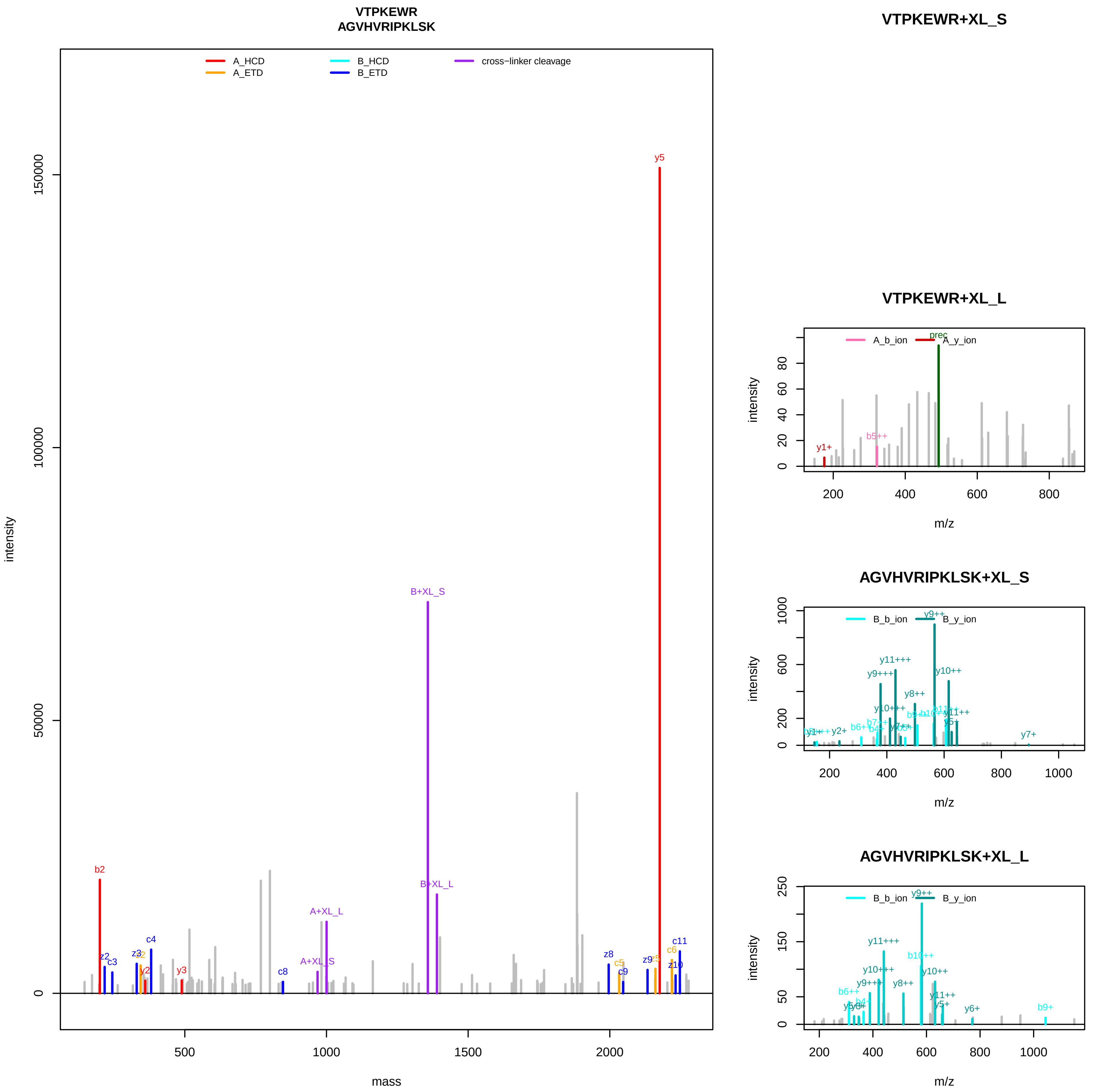

VTPKEWR
AGVHVRIPKLSK
A_HCD
A_ETD
B_HCD
B_ETD
cross−linker cleavage
y5
B+XL_S
B+XL_L
A+XL_L
A+XL_S
b2
c4
z2
c3
z3
y2
y3
c8
z8
c5
c9
z9
c6
c11
z10
intensity
mass
0
50000
100000
150000
500
1000
1500
2000
VTPKEWR+XL_S
VTPKEWR+XL_L
A_b_ion
A_y_ion
prec
b5++
y1+
AGVHVRIPKLSK+XL_S
B_b_ion
B_y_ion
y9++
y11+++
y9+++
y10++
y8++
y10+++
y7+
y2+
AGVHVRIPKLSK+XL_L
y11+++
b10++
y10+++
y10++
y9+++
y8++
b6++
y11++
y5+
y6+
b9+
m/z
